# Laparoscopic Resection of an Isolated Myometrial Metastasis From Ileocecal Cancer: A Case Report

**DOI:** 10.7759/cureus.75580

**Published:** 2024-12-12

**Authors:** Toru Hachisuga, Tsuyoshi Fujii, Kenshiro Araki, Jynya Kizaki, Shinji Mizuochi

**Affiliations:** 1 Obstetrics and Gynecology/Gynecologic Oncology, Social Insurance Tagawa Hospital, Tagawa, JPN; 2 Surgical Gastroenterology, Social Insurance Tagawa Hospital, Tagawa, JPN; 3 Pathology, Social Insurance Tagawa Hospital, Tagawa, JPN

**Keywords:** colon cancer recurrence, diagnostic laparoscopy, female genital tract, solitary metastasis, systemic chemotherapy

## Abstract

A 67-year-old woman was diagnosed with ileocecal cancer presenting with intestinal obstruction. She underwent an ileocecal resection and D3 lymph node dissection. Pathological diagnosis showed a moderately differentiated adenocarcinoma, pT4aN0M0. Adjuvant chemotherapy using oxaliplatin and capecitabine was administered for six months. A CT scan one year after the initial operation revealed a myometrial nodule measuring 3 cm in diameter as a new lesion. We resected the myometrial nodule using laparoscopy, and no peritoneal metastases were observed. The intraoperative frozen section was suggestive of myometrial metastasis originating from colon cancer. Peritoneal washing cytology was negative for malignancy. Subsequently, a laparoscopic total hysterectomy and bilateral salpingo-oophorectomy were performed. The results of immunohistochemical stains were consistent with the patient’s known colon cancer. The resected uterus, ovaries, and fallopian tubes were found to be free of metastatic colon cancer. We diagnosed the uterine lesion as an isolated myometrial metastasis from colon cancer and proceeded with surveillance without additional adjuvant treatment. She is alive and well one year after the second operation. When uterine metastasis from colon cancer is uncertain on cross-sectional imaging, laparoscopy is a useful tool for making an accurate diagnosis and exploring other intraperitoneal lesions.

## Introduction

According to Global Cancer Statics 2022, the number of new cases of colorectal cancer worldwide was more than 1.9 million, ranking third after breast cancer and lung cancer. In addition, the number of deaths from colorectal cancer was 904,000, ranking second after lung cancer [1]. While the prognosis of early-stage colorectal cancer was relatively good, with a five-year survival rate of 91.6% for stage I disease, that of metastatic colorectal cancer was remarkably poor; in patients with stage IV disease, it is only 18.8% [2]. Colorectal cancer commonly metastasizes to the liver, lungs, peritoneum, and lymph nodes. Distant metastases through the blood and lymphatic stream mostly occur in the liver and lung [3]. Metastases from extragenital tumors to the female genital tract are uncommon and usually affect the ovaries [4] [5]. Although the reason for the rarity of this occurrence remains unclear, several possible causes have been postulated including the centrifugal drainage of lymphatics from the uterus and the fibrous nature of cervical stroma [6]. Direct extension of colorectal cancer is considered the most common pathway for secondary involvement of the uterus, usually in the context of a widely disseminated disease [6]. In cases with direct invasion or peritoneal dissemination, uterine metastasis probably occurred on the uterine serosa, subsequently invading the myometrium, while noncontiguous, hematogenous, or lymphatic spread is exceptional among cases in which a widely disseminated disease is not apparent [7]. Herein, we reported a case of isolated myometrial metastasis from ileocecal cancer.

## Case presentation

**Figure 1:**
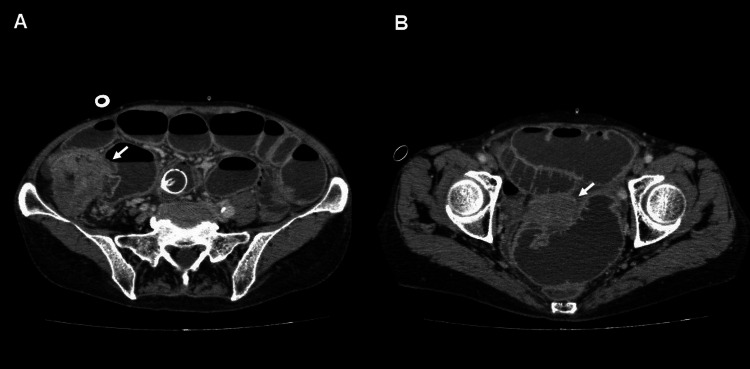
Preoperative CT findings. (A) Ileocecal cancer measuring 7 cm in diameter (arrow), causing intestinal obstruction. An ileus tube was placed. (B) Normal-sized uterus (arrow).

A 67-year-old woman was diagnosed with ileocecal cancer presenting with intestinal obstruction, without any lesions in the female genital tract (Figures 1A-1B).

**Figure 2:**
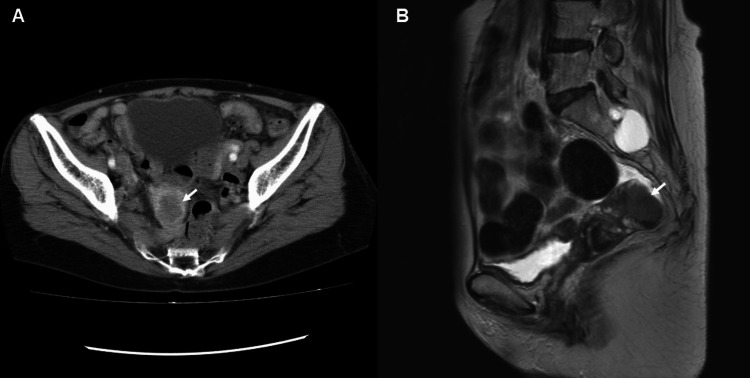
The images one year after the initial operation. (A) A CT image showing a myometrial nodule measuring 3 cm in diameter (arrow). (B) A T2-weighted (T2W) MRI image showing a myometrial nodule with a slightly high signal (arrow).

Tumor makers were within normal range, including carcinoembryonic antigen (CEA) and carbohydrate antigen 19-9 (CA19-9). She underwent an ileocecal resection and D3 lymph node dissection. The findings during open abdominal surgery included a severely distended small intestine without adhesions and an ileocecal tumor that adhered to the retroperitoneum but was free from the female genital organs. Pathological diagnosis showed a moderately differentiated adenocarcinoma, pT4aN0M0. Adjuvant chemotherapy using oxaliplatin and capecitabine was administered for six months. During follow-up, a CT scan from the chest to the pelvis, performed one year after the initial operation, revealed a new myometrial nodule measuring 3 cm in diameter (Figure 2A). She was referred to the gynecology department, presenting no clinical symptoms, including vaginal bleeding. The cervical Papanicolaou smear and endometrial cytology were both negative for malignancy. MRI revealed a 3 cm diameter nodule in the myometrium, which appeared as a low signal on T1-weighted (T1W) images and a slightly high signal on T2-weighted (T2W) images, isolated from the endometrium (Figure 2B).

**Figure 3:**
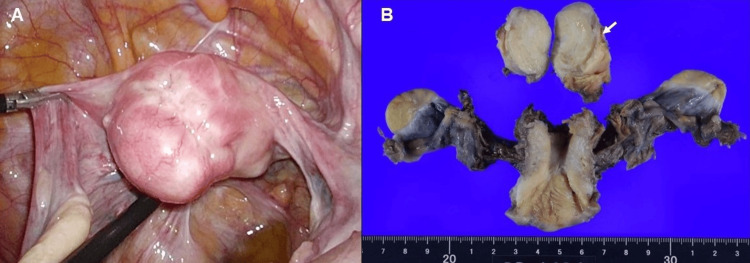
Laparoscopic findings and surgical specimens. (A) Laparoscopy showing a myometrial nodule with an ulcerative scar on the uterine surface. (B) The laparoscopically resected myometrial nodule (arrow) along with specimens from the subsequent laparoscopic total hysterectomy and salpingo-oophorectomy.

The MRI diagnosis suggested a degenerated leiomyoma, but the possibility of malignancy was not completely excluded. Laparoscopy showed a myometrial nodule with an ulcerative scar on the uterine surface (Figure 3A). There were a few intraperitoneal adhesions after ileocecal resection. No metastatic peritoneal lesion was observed. A myometrial nodule was resected laparoscopically. The intraoperative frozen section was suggestive of uterine metastasis from colon cancer. The laparoscopic total hysterectomy and bilateral salpingo-oophorectomy were subsequently performed (Figure 3B).

**Figure 4:**
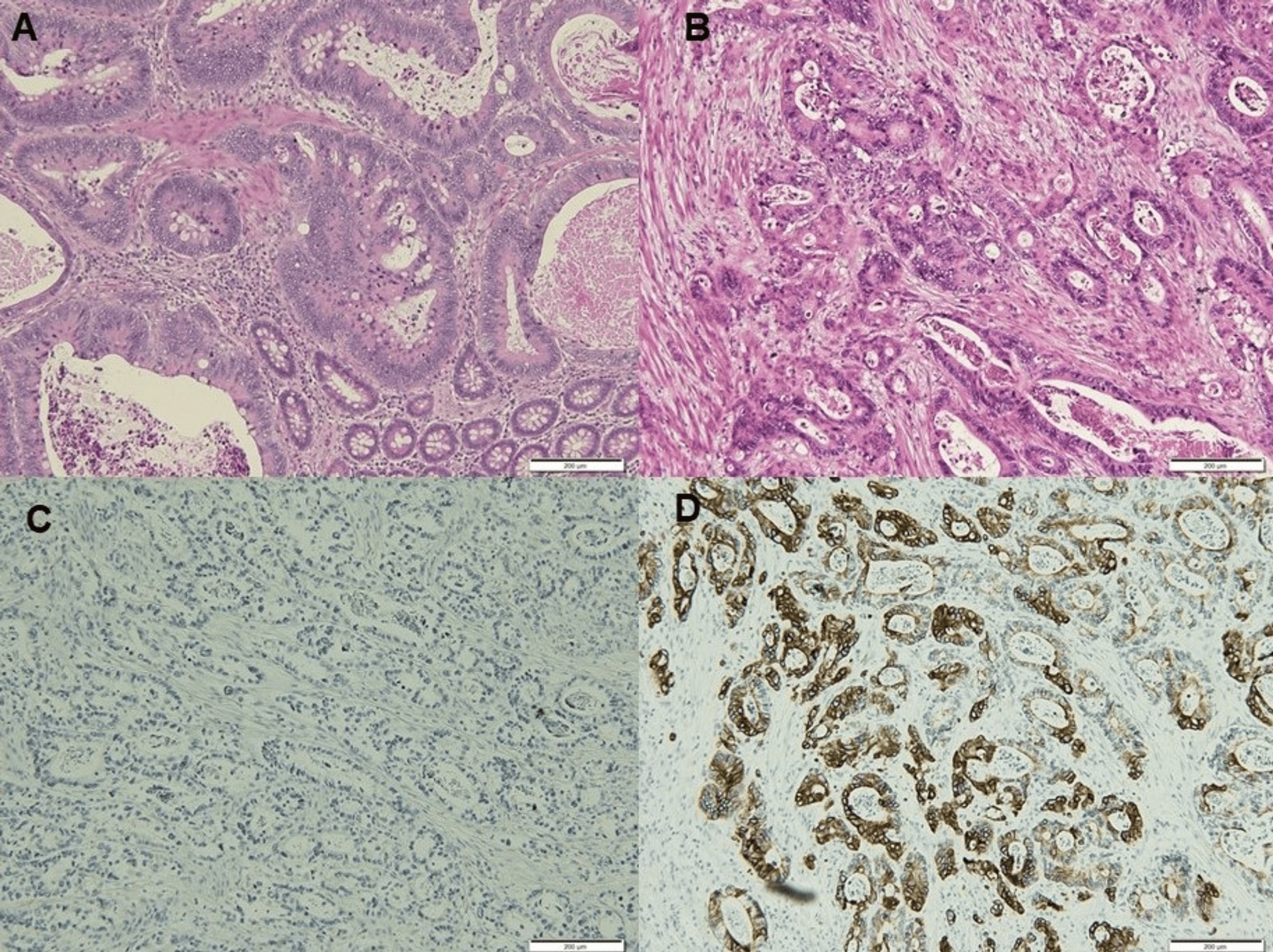
Histopathologic and immunohistochemical findings. (A) The primary ileocecal cancer (hematoxylin and eosin stain). (B) The myometrial nodule (hematoxylin and eosin stain) showing (C) negative for CK7 and (D) positive for CK20.

The operation time was 2 hours and 46 minutes, which included the laparoscopic resection of the myometrial nodule, intraoperative pathological diagnosis, and laparoscopic total hysterectomy with bilateral salpingo-oophorectomy. The perioperative blood loss was 10 mL. She had seven days hospitalization without postoperative complications. The immunohistochemical stains revealed tumor cells of the myometrial nodule to be positive for keratin 20 (CK 20) and negative for keratin 7 (CK 7) and estrogen receptor (Figure 4).

These results were consistent with the patient’s known colon cancer. The peritoneal washing cytology was negative for malignancy. The subsequently resected uterus, ovaries, and fallopian tubes were free from metastatic colon cancer. We diagnosed the myometrial lesion as an isolated myometrial metastasis from colon cancer and followed up with surveillance without adjuvant treatment after the second operation. She is alive and well one year after the second operation, with no evidence of recurrence on the CT scan.

## Discussion

Colon cancer commonly metastasizes to the liver, lungs, peritoneum, and lymph nodes [3]. The corpus uteri metastases from colon cancers were very rare. The majority of metastases to the corpus uteri from extragenital cancers involve the myometrium and are associated with synchronous ovarian metastasis [8]. A case report suggested that myometrial metastases may represent tumor spread from exfoliated ovarian tumor cells [7]. In such instances, serosal tumor deposits should be present either macroscopically or microscopically, along with the involvement of the outer third of the myometrial wall [7]. In the present case, an ulcerative scar on the uterine surface was observed, but serosal tumor deposits were not present either macroscopically or microscopically. The ovaries and fallopian tubes were free from metastatic colon cancer. The peritoneal washing cytology was negative for malignancy. We cannot decide the pathway leading from colon cancer to uterine metastasis.

The study included 296 patients with ovarian metastasis from colorectal cancer [9], and the estimated incidence of ovarian metastasis was 1.4%. The 3-year overall survival rate was 68.6% for solitary ovarian metastases. Peritoneal metastasis occurred more frequently in the cases of synchronous ovarian metastasis than those of metachronous ovarian metastasis. Peritoneal metastasis was an independent unfavorable prognostic factor for the patients with ovarian metastasis from colorectal cancer.

Patients with peritoneal metastasis from colon cancer have reduced overall survival compared with patients with metastatic colon cancer without peritoneal involvement [10]. Laparoscopy is less invasive and useful for searching peritoneal metastasis to be undetected by cross-section imaging [11]. Laparoscopy is considered one of the useful tools for determining whether uterine metastasis from colon cancer is isolated.

The patient in this case is alive and well one year after the second operation, without receiving adjuvant chemotherapy. Despite improvements in systemic chemotherapy, no improvement in the prognosis of the patients with ovarian metastasis from colorectal cancer was observed [9]. A phase II or III randomized controlled trial (hepatectomy followed by mFOLFOX6 versus hepatectomy alone for liver-only metastatic colorectal cancer) suggested that it remains unclear whether chemotherapy improves overall survival [12]. In the literature, the isolated uterine metastasis from colon cancer was reported in four case reports [4,5,13,14]. One patient was treated with adjuvant chemotherapy after hysterectomy [14]. We could not find a multicase study on isolated uterine metastasis from colon cancer. The number of cases is too small to evaluate the effect of adjuvant chemotherapy for isolated uterine metastasis from colon cancer.

Histologically, the differential diagnosis includes endometrioid endometrial carcinoma. In the present case, the myometrial nodule was free from atrophic endometrium. There was no hyperplasic and/or carcinomatous lesion in the endometrium. The immunohistochemical expressions of myometrial nodule were positive for CK20 and negative for CK7 and estrogen receptor. This evidence supports the diagnosis of metastatic carcinoma of colon origin [15].

## Conclusions

When uterine metastasis from colon cancer is identified, it is prognostically important to determine whether the uterine metastasis is isolated. Laparoscopy is less invasive and is useful for detecting peritoneal metastasis. If laparoscopic findings indicate isolated uterine metastasis, the laparoscopic hysterectomy and bilateral salpingo-oophorectomy may be considered a curative method. The benefits of adjuvant chemotherapy after curative resection for metastatic colon cancer are still unclear.
